# Dual inhibition of Type I and Type III PI3 kinases increases tumor cell apoptosis in HER2+ breast cancers

**DOI:** 10.1186/s13058-015-0656-2

**Published:** 2015-12-04

**Authors:** Christian D. Young, Carlos L. Arteaga, Rebecca S. Cook

**Affiliations:** Department of Medicine, Vanderbilt University, 2220 Pierce Avenue, Nashville, TN 37232 USA; Department of Cancer Biology, Vanderbilt University, 2220 Pierce Avenue, Nashville, TN 37232 USA; Department of Breast Cancer Research Program, Vanderbilt Ingram Cancer Center, 2220 Pierce Avenue, Nashville, TN 37232 USA

## Abstract

**Introduction:**

Human epidermal growth factor receptor-2 (HER2) gene amplification (HER2+) drives tumor cell growth and survival in ~25 % of breast cancers. HER2 signaling activates the type I phosphoinositide 3-kinase (PI3K), upon which these tumors rely. Consequently, inhibitors of HER2 and type I PI3K block growth and increase apoptosis in HER2+ breast cancers, especially when used in combination. However, the impact of type III PI3K inhibition, particularly in combination with HER2 blockade or type I PI3K inhibition, remains less clear.

**Methods:**

We utilized small molecule kinase inhibitors, locked nucleic acid antisense oligonucleotides (LNA-ASOs), and siRNA to assess proliferation, autophagy, apoptosis, and protein expression in cell culture models of HER2+ breast cancers.

**Results:**

Treatment of HER2+ breast cancer cells with HER2 inhibitors or type I PI3K kinase inhibitors, alone or in combination, blocked type I PI3K signaling, reduced tumor cell growth, and induced autophagy. Knockdown of the type I PI3K, p110α, using an LNA-ASO termed EZN4150 inhibited PI3K-mediated Akt phosphorylation. However, in contrast to catalytic inhibitors of type I PI3Ks, EZN4150 did not induce autophagy, and blocked autophagy in response to inhibitors of HER2 or type I PI3Ks in a dominant fashion. Sequence analysis of EZN4150 revealed significant homology to the gene encoding the type III PI3K, Vps34, a key component for autophagy induction. EZN4150 simultaneously reduced expression of both p110α and Vps34. Combined inhibition of PI3K signaling and autophagy using individual siRNAs against p110α and Vps34 or using pharmacological type I and type III PI3K inhibitors recapitulated what was seen with EZN4150, and robustly enhanced tumor cell killing.

**Conclusions:**

These studies highlight the important role of Vps34-mediated autophagy in limiting the anti-tumor response to inhibitors of HER2 or type I PI3K in HER2+ breast cancers. The type III PI3K Vps34 represents a potential therapeutic target to block treatment-induced autophagy and enhance tumor cell killing.

**Electronic supplementary material:**

The online version of this article (doi:10.1186/s13058-015-0656-2) contains supplementary material, which is available to authorized users.

## Introduction

Breast cancer afflicts over one million individuals, causing death in nearly a half million people worldwide every year [1]. Breast cancer is subdivided into three clinical subtypes: estrogen receptor positive (ER+), human epidermal growth factor receptor-2 (HER2) positive (HER2+) and triple negative. Because ER and HER2 are important drivers of breast cancer, molecularly targeted therapies against these proteins and their signaling pathways are approved for treatment of patients with these cancer subtypes.

The HER2 tyrosine kinase (RTK) heterodimerizes with a related RTK, ErbB3, to activate several signal transduction pathways, including the type I phosphoinositide 3-kinase (PI3K) pathway, specifically the p110α catalytic subunit of PI3K [2]. Conditional gene targeting of ErbB3 or p110α in the mammary gland abrogates HER2-mediated tumor formation in genetically engineered mice [3, 4]. Similarly, loss of Akt1, a key p110α effector, impairs HER2-induced mammary tumorigenesis in mice, underscoring the importance of the effectors downstream of HER2 and p110α [5, 6]. In contrast, the p110β isoform of type I PI3K plays a dominant role in phosphatase and tensin homolog (PTEN)-null breast tumors, but is not required for growth in many HER2-amplified breast cancers [7]. Importantly, type I PI3K inhibitors potently reduce growth of HER2+ tumor cells in culture and in vivo, and combinations of type I PI3K and HER2 inhibitors display superior anti-tumor activity against HER2+ cancers, suggesting that multiple signaling nodes in the HER2/PI3K pathway require inhibition to abrogate feedback PI3K re-activation that often occurs in response to single-agent inhibition [8–13].

A key downstream effector of PI3K/Akt is mTOR, a kinase that increases cellular energy consumption to drive anabolic processes including protein translation and lipid synthesis to support tumor cell proliferation [14]. In contrast, catabolic processes like autophagy are activated in response to lowered cell energetics under the regulation of AMPK, which phosphorylates ULK1, Vps34/class III PI3K and other regulatory factors [15]. By competing with AMPK for phosphorylation of autophagy regulatory factors, mTOR reduces autophagy [16–18]. Conversely, autophagy is induced upon blockade of mTOR or factors upstream of mTOR, transiently supporting cell survival and reducing the anti-tumor impact of therapeutic inhibitors of HER2, type I PI3Ks, and mTOR [17, 19]. Tumor cell death in response to mTOR inhibition is enhanced by the use of autophagy inhibitors [20–23].

Herein, we demonstrate that combined targeting of type I (p110α) and type III (Vps34) PI3Ks using a single locked nucleic acid antisense oligonucleotide (LNA-ASO) sequence with homology to both transcripts, or using pharmacological inhibitor to each, attenuated signaling through Akt/mTOR, yet prevented autophagy induction typically seen upon mTOR inhibition. As a result, combined inhibition of p110α and Vps34 markedly increased tumor cell killing, and improved tumor growth inhibition in response to HER2 inhibitors. Our results support the utility of inhibiting autophagy when using HER2 or PI3K inhibitors and identify a single oligonucleotide sequence capable of simultaneous ablation of both p110α and Vps34.

## Methods

### Reagents

Commercially purchased antibodies and siRNA are listed in Table 1. Cell culture reagents including fetal bovine serum (FBS) were purchased from Life Technologies (Grand Island, NY, USA). EZN4150 (targeting p110α) and EZN3046 (non-targeting scrambled control for EZN4150) were provided by Enzon pharmaceuticals (Piscataway, NJ, USA). BYL719 and BKM120 were provided by Novartis (Cambridge, MA, USA). Lapatinib was purchased from LC laboratories (Woburn, MA, USA). SAR405 was purchased from ApexBio (Houston, TX, USA). pBABEpuro GFP-LC3 was a gift from Jayanta Debnath (Addgene plasmid # 22405).

**Table 1:** Antibodies and siRNAs

Antibody	Vendor	Catalog number	Dilution	siRNA	Vendor	Catalog number
p110α	Cell Signaling	4249	1:1,000	Universal neg ctrl	Sigma	SIC003
p110β	Cell Signaling	3011	1:1,000	p110α-silencing	Sigma	SASI_Hs01_002193**38**
P-Akt (S473)	Cell Signaling	9271	1:1,000	p110α-silencing	Sigma	SASI_Hs01_002193**39**
Akt	Cell Signaling	9272	1:2,000	Vps34-silencing	Sigma	SASI_Hs01_002337**16**
P-S6 (S235/6)	Cell Signaling	4858	1:1,000	Vps34-silencing	Sigma	SASI_Hs02_003341**79**
S6	Cell Signaling	2317	1:1,000			
P-HER2 (Y1221)	Cell Signaling	2243	1:1,000			
HER2	Thermo	Rb103	1:1,000			
Vps34	Cell Signaling	3358	1:1,000			
LC3	Cell Signaling	4108	1:1,000			
Actin	Cell Signaling	4970	1:5,000			
PARP	Cell Signaling	9532	1:1,000			
Cleaved caspase 3	Cell Signaling	9661	1:500			

*HER2* human epidermal growth factor receptor-2, *PARP* poly(ADP-ribose) polymerase Cell Signaling Technologies (Danvers, MA, USA). Thermo-Fisher (Waltham, MA, USA). Sigma-Aldrich (St. Louis, MO, USA)

### Cell culture, siRNA transfection and virus production

All cells were obtained from ATCC (Manassas, VA, USA) and maintained in DMEM supplemented with 10 % FBS. LNA-ASO compounds were applied to cells at 5 μM (unless otherwise noted) without transfection reagent, replenishing in fresh media every 3–4 days in studies exceeding four days. siRNA complexes were prepared at 500 nM in OptiMEM and diluted 10-fold into culture media for a final concentration of 50 nM. For example, 100 μl of 500 nM siRNA was prepared by mixing 2.5 μl of 20 μM siRNA and 3 μl Lipofectamine RNAiMAX in a final volume of 100 μl OptiMEM, allowing complexes to form for 15 minutes and then applying them to cells in 900 μl of media for a final 50 nM siRNA. When two siRNAs were combined, each was applied at a final concentration of 50 nM and non-targeting control siRNA was used in combination with targeting siRNA such that all cells received a total of 100 nM siRNA. Amphotropic retrovirus was generated by transfecting 24 μg proviral plasmid into Phoenix-Ampho cells in 10-cm dishes using 60 μl Lipofectamine 2000. Packaging cells were fed 24 h post-transfection; virus-containing supernatants were harvested 48 and 72 h post-transfection, diluted 1:4 and applied to target cells with 8 μg/ml polybrene. Target cells were selected with 1 μg/ml puromycin.

### Proliferation, caspase 3/7 and annexin-V assays

Proliferation assays were performed by plating 5 × 10^4^ cells/well of 6-well plates or 2 × 10^4^ cells/well of 12-well plates and treating with LNA-ASO compounds as indicated. Fresh media and compounds were applied every 3 days. Cells from triplicate wells of each treatment group were trypzinised and counted with a TC10 hemocytometer (Biorad; Hercules, CA, USA) on the indicated days. Alternatively, 5 × 10^3^ cells/well were seeded in 96-well plates, treated as described and relative proliferation determined by sulforhodamine B (SRB) assay or DNA quantification. Briefly, media were decanted and monolayers fixed with 10 % TCA, washed with tap water, dried, stained with 0.4 % SRB in 1 % acetic acid, de-stained with 1 % acetic acid, dried, solubilized with 10 mM Tris base and quantitated by spectrophotometric detection at 490 nm using a plate reader. Proliferation is presented relative to the control treated cells or the day-0 value. For caspase 3/7 activity and annexin V staining, cells seeded in 96-well plates were pretreated for 30 minutes with dimethyl sulfoxide (DMSO) control or 1 μM SAR405 30 minutes before being treated with DMSO control, 0.5 μM BYL719 or 0.5 μM lapatinib. Cells were lysed with an equal volume of caspase 3/7 reagent (Promega; Fitchburg, WI, USA) after the indicated time point (3, 6, 12 or 24 h), incubated for 45 minutes and luminescent caspase 3/7 activity was assessed on a 96-well plate luminometer. For annexin V staining, cells were treated similarly (BT474 and UACC893 for 6 h, SKBR3 for 24 h) with 5 μl of AlexaFluor 594-conjugated annexin V added 1 h and 1 μg/ml Hoescht 33342 added 15 minutes before the end of treatment. Images were captured with an ImageExpress 96-well plate fluorescent microscope (Molecular Devices; Sunnyvale, CA, USA) with nine × 10 images captured per well of quadruplicate wells. The total number of nuclei and number of annexin V-positive cells was determined with MetaXpress software and is presented as the percentage of annexin V-positive cells.

### Immunoblot

Lysates were generated by removing media from cells, washing monolayers with cold PBS and lysis with RIPA: 50 mM Tris, pH 7.4, 150 mM NaCl, 1 % NP-40, 0.5 % Deoxycholate, 0.1 % SDS, 1 mM EDTA, 50 mM NaF, 1 mM NaVO_4_ and 1 × protease inhibitor cocktail (Roche). Lysates were clarified by centrifugation at 15,000 × g for 15 minutes. Protein concentration was determined by bicinchoninic acid (BCA) assay (Thermo scientific; Waltham, MA, USA). For immunoblot analysis, equal amounts of protein/lane were subjected to SDS-PAGE, transferred to nitrocellulose membranes and analyzed with the antibodies detailed above.

### Statistical analyses

GraphPad Prism was used for statistical analyses. For two-group analyses, the Student *t* test was conducted. In analyses of more than two groups, analysis of variance (ANOVA) was conducted with Tukey multiple comparison analyses to compare individual groups. Error bars represent standard error of the mean (SEM).

## Results

### EZN4150 reduces p110α expression, PI3K signaling and growth in HER2+ breast cancer cells

We used LNA-ASOs to inhibit the PI3K signaling axis in HER2+ breast cancer cells. Unlike kinase inhibitors, which generally block ATP-dependent enzymatic activity, ASOs bind to and downregulate transcripts encoding any chosen gene product, including “non-druggable” targets (e.g., adaptor molecules, transcription factors, or cancer-specific mutant transcripts) [24, 25]. For example, EZN3920 is an ErbB3-directed LNA-ASO which potently inhibits growth and survival of HER2+ tumor cells both in vitro and in vivo [25–27]. We used EZN4150, an LNA-ASO directed against the *PIK3CA* gene, encoding the p110α type I PI3K catalytic subunit. HER2+ breast cancer cells (MDA-MB-361, BT474 and SKBR3) were treated with escalating doses of EZN4150 or with EZN3046, a scrambled non-targeting control LNA-ASO. EZN4150 produced a dose-dependent reduction in p110α expression in all three cell lines, with maximal p110α knock-down at 5 μM (Additional file 1: Figure S1A). EZN4150 reduced expression of p110α but not p110β, demonstrating the isoform specificity of EZN4150 (Additional file 1: Figure S1B). EZN4150 reduced P-Akt in MDA-MB-361, BT474 and SKBR3 cells, but did not affect P-Akt in PTEN-deficient HCC1937 cells, consistent with previous observations that PTEN-deficient breast cancer cells preferentially rely on p110β, not p110α, for type I PI3K signaling [3, 7].

**Fig. 1:**
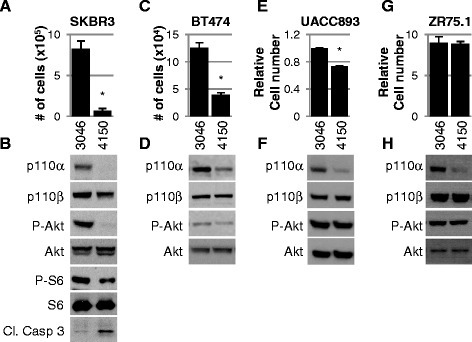
EZN4150 inhibits proliferation of human epidermal growth factor receptor-2 positive (HER2+) breast cancer cells. **a**, **b** SKBR3 cells were treated with the 5 μM EZN3046 (scrambled control locked nucleic acid antisense oligonucleotide (LNA-ASO)) or EZN4150 (p110α targeted LNA-ASO). **a** Proliferation was assessed on day 12 of treatment. **b** Lysates were prepared for immunoblot analysis on day 9. **c**, **d** BT474 cells were treated as shown in **a** and **b**. Cells were counted (**c**) or lysed for immunoblot analysis (**d**) after 10 days of treatment. **e**, **f** UACC893 cells were treated as shown in panels **a** and **b**. **e** Relative cell number was determined on day 16 of treatment by sulforhodamine B assay. **f** Cells were lysed for immunoblot analysis after 12 days of treatment. **g**, **h** ZR75.1 cells were treated with the 5 μM EZN3046 or EZN4150. **g** Relative proliferation was determined by quantifying DNA in each well after 12 days of treatment. **h** After 10 days of treatment, cells were lysed and analyzed by immunoblot analysis (*p <0.05, Student *t* test). *Cl. Casp3* cleaved caspase 3

We examined the impact of EZN4150 on growth of SKBR3, BT474 and UACC893 HER2+ cells. EZN4150 treatment for 12 days decreased SKBR3 cell numbers >10-fold as compared to EZN3046 treatment (Fig. 1a), while decreasing phosphorylation of Akt (Fig. 1b), thus demonstrating inhibition of PI3K signaling. Decreased phosphorylation of S6, a distal effector of mTOR signaling, was also observed in SKBR3 cells treated with EZN4150. Increased caspase 3 cleavage in response to EZN4150 suggested that targeted downregulation of p110α using EZN4150 may induce tumor cell death. Similarly, EZN4150-mediated p110α downregulation significantly decreased the number of BT474 cells after 10 days of treatment, and decreased Akt phosphorylation (Fig. 1c-d). The HER2+ cell line UACC893 displayed modest but significant diminution of tumor cell numbers after 16 days in culture with EZN4150. Interestingly, Akt phosphorylation was not impacted by EZN4150 in UACC893 cells, despite robust p110α downregulation, perhaps explaining the decreased responsiveness of UACC893 cells to EZN4150 relative to BT474 or SKBR3 (Fig. 1e-f). In contrast, PTEN-null ZR75.1 cell numbers and P-Akt were not impacted by EZN4150 (Fig. 1g-h), consistent with the idea that PTEN-null cells preferentially use p110β for Akt activation and cell survival.

### Inhibition of tumor cell growth in response lapatinib and BKM120 is enhanced by EZN4150

**Fig. 2:**
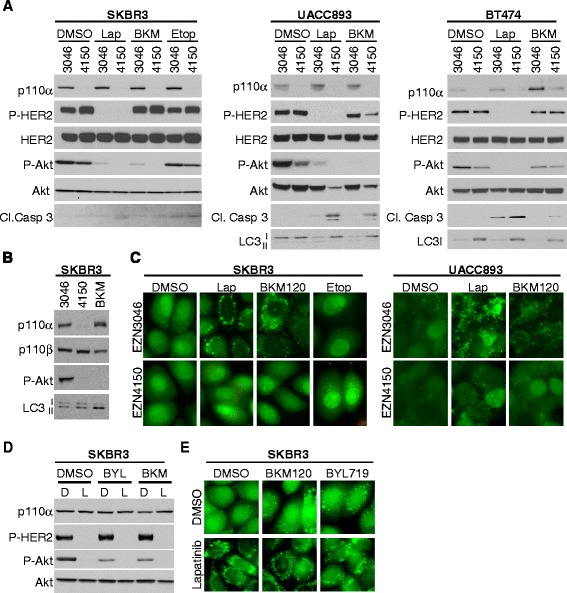
The inhibition of human epidermal growth factor receptor-2 positive (HER2+) breast cancer cells in response to lapatinib and BKM120 is enhanced by EZN4150. **a** SKBR3 were cultured for 6 days, UACC893 cells were cultured for 7 days and BT474 cells were cultured for 9 days with 5 μM EZN3046 or EZN4150. In the final 12 h, dimethyl sulfoxide (*DMSO*), 1 μM Lapatinib (*Lap*), 1 μM BKM120 (*BKM*) or 50 μM etoposide (*Etop*) was added. Lysates were harvested and analyzed by immunoblot. **b** SKBR3 cells were cultured for 6 days with 5 μM EZN3046 or EZN4150 or for 3 days with 500 nM BKM120 (*BKM*). Lysates were harvested and analyzed by immunoblot with the indicated antibodies. **c** SKBR3 GFP-LC3 cells and UACC893 GFP-LC3 cells were cultured as described in **a**. Green fluorescent protein (GFP)-LC3 (*green*) localization was captured by fluorescent microscopy. **d**, **e** SKBR3 GFP-LC3 cells were cultured for 5 days with DMSO, 500 nM BKM120 or 500 nM BYL719. Cells were treated with DMSO (*D*) or 1 μM Lapatinib (*L*) for the final 18 h. **d** Lysates of cells were evaluated by immunoblot analysis with the indicated antibodies. **e** GFP-LC3 localization was captured by fluorescent microscopy

To determine the impact of EZN4150-mediated p110α ablation on HER2+ tumor cell response to the EGFR/HER2 tyrosine kinase inhibitor (TKI) lapatinib [28], we treated SKBR3, BT474, and UACC893 cells with EZN4150 in the presence or absence of lapatinib. While lapatinib induced low levels of caspase 3 cleavage in all three cell lines, the addition of EZN4150 increased cleaved caspase 3 and decreased P-Akt to a greater extent than was seen with either agent alone (Fig. 2a). Like EZN4150-mediated p110α ablation, catalytic inhibition of PI3K using the pan-type I PI3K inhibitor BKM120 [29] decreased P-Akt in all three cell lines, but did not induce cleavage of caspase 3. However, EZN4150 in combination with BKM120 decreased P-Akt to a greater degree than either EZN4150 or BKM120 alone, and increased cleaved caspase 3 levels to a greater extent than either single compound in all three cell lines, suggesting that the effects of EZN4150 are not entirely identical to the effects achieved using catalytic PI3K inhibition. Further, these results suggest that EZN4150 enhanced lapatinib-mediated or BKM120-mediated caspase 3 activation.

Inhibition of PI3K/mTOR signaling is known to induce autophagy, a process in which cytoplasmic contents are degraded in order to maintain cellular energy balance and survival [16, 20]. Consistent with this idea, BKM120 induced autophagy in SKBR3 cells, as indicated by the decreased presence of the higher molecular-weight species of LC3 (LC3I) (Fig. 2b), which is modified to a lower molecular-weight species (LC3II) and subsequently degraded upon induction of autophagy. Similarly, LC3I diminution was seen in UACC893 and BT474 cells treated with BKM120 as compared to control-treated cells (Fig. 2a). Lapatinib similarly induced diminution of LC3I in UACC893 and BT474 cells. However, cells treated with EZN4150 did not display LC3I diminution, even in the presence of BKM120 or lapatinib (Fig. 2a, b). These results thus distinguish the effects of p110α-targeting using EZN4150 from those using BKM120. Specifically, these results suggest that autophagy is induced in response to HER2 inhibition and/or type I PI3K inhibition, but is blocked by EZN4150, despite its ability to block type I PI3K kinase activity.

These results were confirmed in SKBR3 and UACC893 cells expressing green fluorescent protein (GFP)-tagged LC3, which will accumulate to form GFP+ puncta on the surface of autophagosomes once autophagy is induced. HER2/PI3K inhibition with lapatinib increased GFP-LC3 puncta in SKBR3 and UACC893 cells (Fig. 2c). Similarly, LC3-GFP puncta were seen in cells treated with BKM120, but not with etoposide, demonstrating the specificity of autophagy induction to PI3K pathway blockade rather than to a generalized induction of cell death. In contrast to what was seen with BKM120-mediated inhibition of type I PI3K signaling, EZN4150 used as a single agent did not induce the formation of GFP-LC3 puncta. Further, EZN4150 blocked LC3-GFP+ autophagosome formation in lapatinib-treated cells and in BKM120-treated cells, again suggesting that EZN4150 exerts an impact on HER2+ cells that is separable from what is achieved by the catalytic type I PI3K inhibitor.

Because EZN4150 requires extended treatment to achieve p110α knockdown, it is possible that EZN4150 inhibits autophagy through prolonged downregulation of p110α (5–10 days), as opposed to acute inhibition (12–24 h) of catalytic type I PI3K activity by BKM120 or lapatinib. However, even after 5 days of treatment with BKM120 or BYL719 (a p110α-specific PI3K inhibitor [30]) to produce sustained inhibition of PI3K signaling through the type I PI3K pathway (Fig. 2d), GFP-LC3+ autophagosomes were abundant as compared to what was seen in cells treated with DMSO (Fig. 2e). Lapatinib treatment for the final 24 h of culture further increased LC3-GFP+ autophagosomes in SKBR3 cells pre-treated for 5 days with BKM120 or BYL719. These findings suggest that both acute and sustained PI3K inhibition induces autophagy, and that prolonged PI3K inhibition did not exhaust or reduce autophagosome formation.

### Dual inhibition of p110α and Vps34 by EZN4150

**Fig. 3:**
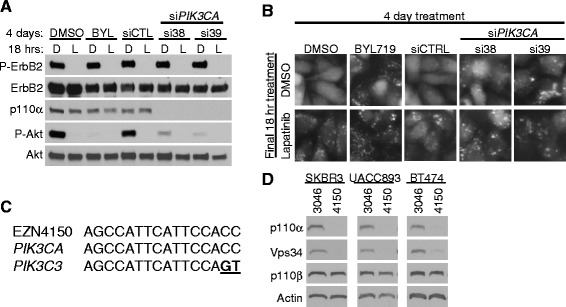
EZN4150 inhibits p110α and Vps34. **a**, **b** SKBR3 green fluorescent protein (GFP)-LC3 cells were transfected with 50 nM control non-targeting siRNA (*siCTL*) or two siRNA sequences targeting *PIK3CA* (*si38* and *si39*) and cultured for 4 days. Parallel plates were cultured for 4 days with dimethyl sulfoxide (*DMSO*) or 500 nM BYL719. Cells were treated with DMSO control (*D*) or 1 μM Lapatinib (*L*) for the final 18 h. **a** Lysates of cells were evaluated by immunoblot analysis with the indicated antibodies. **b** GFP-LC3 localization was captured by fluorescent microscopy. **c** Alignment of the EZN4150 sequence with the sequences of human *PIK3CA* and *PIK3C3*, the genes encoding p110α and Vps34, respectively. Misaligned bases are underlined in bold. **d** Cells were treated with 5 μM EZN3046 or EZN4150 for 7–8 days. Lysates were analyzed by immunoblot with the indicated antibodies

The induction of autophagy by inhibition of the catalytic activity of type I PI3K with BYL719 or BKM120 is consistent with the idea that PI3K/mTOR inhibition relieves mTOR-mediated repression of autophagy. However, the failure of EZN4150 to induce autophagy, despite strong downregulation of type I PI3K signaling, suggested two possible scenarios: 1) p110α has a direct but non-enzymatic role in autophagy induction, (a structural function, for example); or 2) EZN4150 impacts autophagy in a p110α-independent manner. To distinguish these possibilities, we knocked down p110α using siRNA sequences against regions of *PIK3CA* distinct from the sequence targeted by EZN4150 (Additional file 2: Figure S2). Similar to what was seen with EZN4150, downregulation of p110α by two independent siRNA sequences (si38 and si39) reduced P-Akt, as compared to a control siRNA sequence (Fig. 3a). Unlike EZN4150, siRNA-mediated p110α knockdown induced GFP-LC3+ autophagosome formation in SKBR3 cells, similar to what was seen using the catalytic p110α inhibitor BYL719 (Fig. 3b). Further, p110α siRNAs failed to block lapatinib-induced GFP-LC3 autophagosome formation in SKBR3 cells, in contrast to what was seen using EZN4150 in combination with lapatinib. These results suggested that EZN4150 may block autophagy through a mechanism that is independent of p110α downregulation.

Sequence analysis demonstrated that the 16 bp EZN4150 nucleotide sequence matched perfectly (16/16 bp) with the DNA sequence encoding human *PIK3CA*, but also matched 14/16 bp to the human *PIK3C3* gene sequence, which encodes the class III PI3K Vps34. Importantly, Vps34 lipid kinase activity is necessary for induction of autophagy [17] (Fig. 3c). Western analysis demonstrated that EZN4150 downregulated both p110α and Vps34 expression in SKBR3, BT474, and UACC893 cells (Fig. 3d). Thus, the inhibition of autophagy by EZN4150 may be mediated by Vps34 ablation [31], which we explored in additional experiments.

**Fig. 4:**
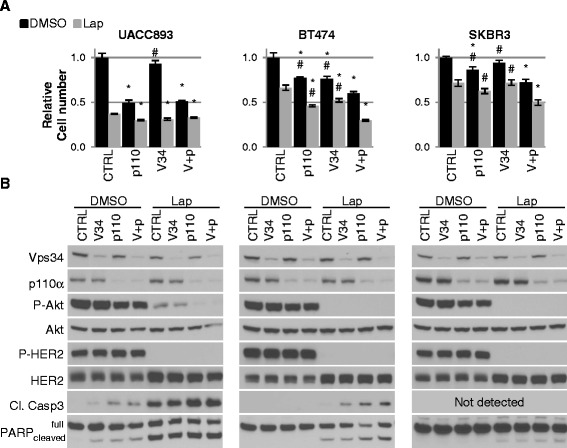
Simultaneous reduction of p110α and Vps34 reduces proliferation and sensitizes human epidermal growth factor receptor-2 positive (*HER2+*) cells to lapatinib. UACC893, BT474 and SKBR3 cells were transfected with siRNA at a final concentration of 100 nM, 50 nM each: 1) siCTRL + siCTRL (*CTRL*); 2) siVps34 + siCTRL(*V34*); 3) sip110α + siCTRL(*p110*); 4) siVps34 + sip110α (*V + p*). Three days post-transfection, cells were treated with dimethyl sulfoxide (*DMSO*) control or 1 μM lapatinib (*Lap*). **a** Relative cell number was assessed 24 h after lapatinib treatment by sulforhodamine B assay. Within each drug treatment (DMSO or Lap): *p <0.05 comparing siCTRL + siCTRL to the other three combinations; #*p* <0.05 comparing single targeted siRNA to siVps34 + sip110; one way analysis of variance with Tukey multiple comparison test. Student *t* test analysis of each DMSO/lapatinib pair was significant (*p* <0.05), and is not graphically depicted. **b** Lysates were harvested 2 h after lapatinib treatment and were analyzed by immunoblot with the indicated antibodies. *PARP* poly(ADP-ribose) polymerase, *C Casp 3* cleaved caspase 3

To determine the combined impact of siRNA-mediated knockdown of Vps34 and p110α, siRNA sequences were used to knock down Vps34 alone, p110α alone, or Vps34 and p110α together. While siRNA sequences targeting p110α inhibited growth of *HER2*-amplified SKBR3, BT474, and UACC893 cells, siRNA sequences targeting Vps34 had little impact on SKBR3 and UACC893 cell growth, and had modest inhibition of BT474 cell growth (Fig. 4a). However, the combined siRNA-mediated knockdown of p110α and Vps34 significantly decreased SKBR3 and BT474 cell numbers more potently than either siRNA alone. Western analysis revealed only partial knockdown of both Vps34 and p110α by the siRNA sequences, inducing only a modest reduction in P-Akt in all three cell lines (Fig. 4b), unlike the robust p110 knockdown and P-Akt reduction seen using EZN4150. Despite only partial knockdown, p110α or Vps34 siRNA increased cleaved caspase 3 and cleaved PARP in UACC893 cells.

The impact of combined p110α and Vps34 knockdown on lapatinib-mediated growth inhibition was examined by treating the cells for the final 24 h of culture with lapatinib. Lapatinib-mediated growth inhibition was observed in SKBR3, BT474, and UACC893 cells, which was enhanced upon knockdown of either p110α or Vps34 in BT474 and UACC893 cells, albeit modestly (Fig. 4a). The combined knockdown of Vps34 and p110α significantly enhanced lapatinib-mediated growth inhibition in BT474 and SKBR3 cells. Increased lapatinib-mediated cleavage of caspase 3 and/or PARP was seen upon combined knockdown of p110α and Vps34 as compared to knockdown of either factor alone in BT474 cells (Fig. 4b). Regardless of siRNA treatment, lapatinib induced high levels of caspase 3 and PARP cleavage in UACC893 cells and very little cleavage in SKBR3 cells. However, slightly increased levels of PARP cleavage were detected in SKBR3 cells with combined knockdown of Vps34 and p110α and treated with lapatinib. These data demonstrate that combined knockdown of p110α and Vps34 using siRNA can induce caspase and PARP cleavage, and enhance lapatinib-mediated growth inhibition in HER2+ breast cancer cells, consistent with what was seen using EZN4150.

### Combined inhibition of HER2, Vps34 and/or p110α induces cell death in HER2+ breast cancer

**Fig. 5:**
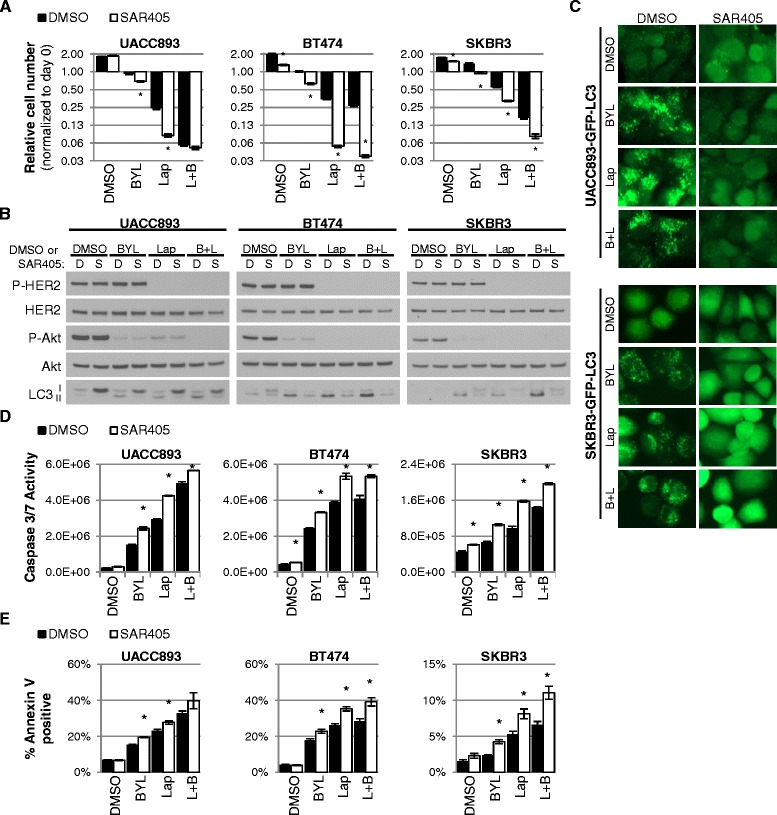
Simultaneous inhibition of human epidermal growth factor recptor-2 (*HER2*), p110α and Vps34 induces cell death in HER2+ breast cancer. **a** UACC893, BT474 and SKBR3 cells were cultured with 1 μM SAR405, 0.5 μM Lapatinib or 0.5 μM BYL719. Relative cell number was determined by sulforhodamine B assay at time 0 and after three days of culture. Day 3 signal divided by day 0 signal was used to normalize cell number to day 0. **b** Cells were treated as described in **a**. Lysates were harvested 6 h after treatment and analyzed by immunoblot analysis with the indicated antibodies. **c** UACC893-GFP-LC3 cells were treated with 1 μM SAR405, 0.5 μM Lapatinib or 0.5 μM BYL719 for 3 h before being fixed and green fluorescent protein (GFP)-LC3 localization determined by fluorescent microscopy. SKBR3-GFP-LC3 cells were analyzed similarly, but fixed after 6 h of treatment. **d**, **e** UACC893 and BT474 cells were treated for 6 h and SKBR3 cells for treated 24 h with 1 μM SAR405, 0.5 μM Lapatinib or 0.5 μM BYL719 before caspase 3/7 activity was determined (**d**) or stained with Annexin V (**e**). In all experiments, SAR405 was applied 30 minutes prior to the application of BYL719, lapatinib or dimethyl sulfoxide (*DMSO*) control (*p <0.05, Student *t* test comparing the effect of DMSO vs. SAR405 within each treatment group)

To confirm these results, we used a small molecular-weight kinase inhibitor of Vps34, SAR405, alone and in combination with inhibitors for p110α and/or HER2. Importantly, Vps34 inhibition using SAR405 has been shown to block the induction of autophagy in response to mTOR inhibition [32]. Similar to what was seen upon siRNA-mediated Vps34 knockdown, Vps34 inhibition using SAR405 had only modest anti-proliferative effects as a single agent in *HER2*-amplified breast cancer cells (Fig. 5a), and no impact on phosphorylation of Akt (Fig. 5b). However, SAR405 enhanced lapatinib-mediated growth inhibition and BYL719-mediated growth inhibition in SKBR3, BT474, and UACC893 cells (Fig. 5a). SKBR3, BT474, and UACC893 cells demonstrated >90 % decrease in relative cell number when lapatinib, BYL719, and SAR405 were used in combination.

The impact of SAR405-mediated Vps34 inhibition on autophagy was examined by western analysis of LC3. While lapatinib and BYL719 each resulted in the diminution of LC3I and/or accumulation of LC3II in the three *HER2*-amplified cell lines tested, SAR405 robustly blocked LC3I diminution and LC3II accumulation (Fig. 5b), consistent with the hypothesis that Vps34 blockade efficiently dampens induction of autophagy upon p110α inhibition. These results were confirmed by fluorescent imaging, examining autophagosomal accumulation of GFP-tagged LC3 in UACC893 and SKBR3 cells. Unlike lapatinib, BYL719, and the combination of lapatinib + BYL719, which induced formation and accumulation of GFP-LC3+ autophagosomes, SAR405 treatment did not cause the formation and accumulation of GFP-LC3+ puncta (Fig. 5c). Further, SAR405 effectively blocked the formation and accumulation of GFP-LC3+ autophagosomes induced by lapatinib, BYL719, or their combination.

To examine the impact of pharmacological Vps34 inhibition on apoptosis, we treated SKBR3, BT474, and UACC893 cells with SAR405 and measured caspase 3/7 activity as a quantitative surrogate for apoptosis. Caspase 3/7 activity was highest after 6 h of treatment in UACC893 and BT474 cells and at 24 h in SKBR3 cells based on time course analyses (Additional file 3: Figure S3A). SAR405 as a single agent modestly but significantly increased caspase3/7 activity in BT474 and SKBR3 cells (Fig. 5d). In all three cell lines, lapatinib treatment robustly induced caspase 3/7 activity, as did BYL719. However, Vps34 inhibition using SAR405 further increased caspase 3/7 activity induced by lapatinib, BYL719 or their combination. These results were confirmed using fluorescence-conjugated Annexin V, which binds to phosphatidyl serine exposed on the outer plasma membrane leaflet of dying cells (Additional file 3: Figure S3B). All cell lines demonstrated <6 % Annexin V-positive cells in the absence of inhibitors. SAR405 used as a single agent did not affect the proportion of cells binding to Annexin V (Fig. 5e), while lapatinib increased the fraction of Annexin V-positive cells to >20 % in UACC893 or BT474 cells and doubled the number of Annexin V-positive SKBR3 cells. Similar to what was seen with lapatinib, BYL719 used as a single agent increased the proportion of Annexin V-positive cells, and the combination of lapatinib + BY719 produced a further increase in the Annexin V-positive cell population in two of three *HER2*-amplified cell lines examined. Notably, inhibition of Vps34 using SAR405 in combination with lapatinib, BYL719, or the combination of lapatinib + BYL719 drove the proportion of Annexin V-positive cells even higher. In summary, the inhibition of Vps34 decreased proliferation and increased cell death induced by either lapatinib or BYL719 in all three HER2+ cell lines, with superior anti-tumor cell responses often observed when HER2, p110α and Vps34 were simultaneously inhibited. These data are consistent with the idea that Vps34 induces autophagy in *HER2*-amplified breast cancer cells in response to type I PI3K pathway blockade as a stop gap survival pathway. As such, inhibition of autophagosome formation using type III PI3K inhibitors (like the Vps34 inhibitor) may enhance tumor cell killing in response to type I PI3K pathway inhibition.

## Discussion

*HER2*-amplified breast cancer cells are exquisitely dependent on the type I PI3K signaling pathway, although the role of type III PI3K is less clear. Combinations of type I PI3K- and HER2-targeted therapeutics are being tested in preclinical cancer models and in clinical trials [11–13, 33–35]. We knocked down the type I PI3K catalytic subunit p110α in *HER2*-amplified breast cancer cells using EZN4150, a locked nucleic acid anti-sense oligonucleotide [3]. EZN4150 has previously been shown to block p110α expression in prostate cancer cell lines [25] and in NSCLC xenografts [27]. We discovered that EZN4150 targets both p110α and Vps34 in a sequence-specific manner, thus inhibiting both class I p110α PI3K signaling and pro-survival autophagy driven by class III PI3K/Vps34 signaling, producing a potent induction of tumor cell death.

As the only conserved member of the PI3K family in all eukaryotes, Vps34 plays a pivotal role in regulating membrane trafficking, including endocytosis, endosome-lysosome maturation, autophagosome formation/flux and cytokinesis [36]. Thus, this ancestral PI3K isoform is critical for nutrient acquisition, energy maintenance and cell division. These functions are partially conserved in class I PI3K of higher eukaryotes, with an example found in cancer cells: PI3K/Akt activity regulates glucose uptake and metabolism, promotes F-actin dynamics during invadopodia formation and tumor cell invasion, and promotes tumor cell survival [37–39]. However, class I PI3K/p110α signaling activates mTOR and suppresses autophagy, thus opposing the activity of class III PI3K/Vps34. This highlights a functional divergence in class I and class III PI3K families in higher eukaryotes [16], which is utilized by tumor cells to promote mTOR-dependent anabolic processes like DNA, lipid and protein synthesis, or to maintain cellular energetics through Vps34-dependent autophagy when mTOR is inhibited [15, 18]. Thus, HER2/PI3K/mTOR inhibitors have greater anti-tumor activity when combined with inhibitors of autophagy [19, 20, 22, 23, 32].

Similar to small molecule type I PI3K inhibitors in other studies [12, 40], EZN4150 sensitized HER2+ breast cancer cells to the HER2 TKI lapatinib. However, unlike small molecule catalytic inhibitors of PI3K, EZN4150 did not induce autophagy, and even blocked the induction of autophagy in response to catalytic type I PI3K inhibitors [17, 31]. Because Vps34 plays a vital role in cellular processes other than autophagy, we cannot rule out that inhibition of other Vps34-dependent pathways contributes to tumor cell apoptosis in response to EZN4150 or SAR405. However, a chemical inhibitor of autophagy flux, chloroquine, has been demonstrated to enhance the effects of HER2 inhibitors [23] suggesting that autophagy is playing a protective role in response to drug treatment. These findings suggest that Vps34-regulated autophagy promotes cell survival upon HER2 or PI3K pathway inhibition, and that blockade of autophagy, using chloroquine or inhibitors of Vps34, improve the antitumor effect of HER2/PI3K pathway inhibitors.

## Conclusions

These studies identify EZN4150 as an antisense oligonucleotide that simultaneously targets p110α, a class I PI3K, and Vps34, the class III PI3K. Targeted inhibition of Vps34 using siRNA-mediated knockdown or pharmacological inhibition blocked the induction of autophagy in response to HER2/PI3K inhibitors, thus increasing therapeutic tumor cell killing in *HER2*-amplified breast cancer cells. These findings warrant future studies examining Vps34 as a therapeutic target to improve tumor cell apoptosis in combination with inhibitors of the HER2/PI3K signaling axis.

## Additional files

Additional file 1: Figure S1. EZN4150 reduces expression of p110α without effecting p110β. (PDF 851 kb) Additional file 2: Figure S2. EZN4150 and p110α siRNA target sequences within the *PIK3CA* gene. (PDF 851 kb) Additional file 3: Figure S3. Combined inhibition of human epidermal growth factor receptor-2 (HER2), p110α and Vps34 induces cell death. (PDF 851 kb)

## Abbreviations

bp: base pair(s)
DMSO: dimethyl sulfoxide
ER: estrogen receptor
DMEM: Dulbecco’s modified Eagle’s serum
GFP: green fluorescent protein
HER: human epidermal growth factor receptor
HER2+: *HER2* gene amplification
LNA-ASO: locked nucleic acid antisense oligonucleotide
PARP: poly(ADP-ribose) polymerase
PBS: phosphate-buffered saline
PI3K: phosphoinositide 3-kinase
*PIK3CA*: gene encoding p110α catalytic subunit of PI3K
*PIK3C3*: gene encoding Vps34 catalytic subunit of type III PI3K
PTEN: phosphatase and tensin homolog
SRB: sulforhodamine B
TKI: tyrosine kinase inhibitor
