# Supplementary material for: Dual inhibition of Type I and Type III PI3 kinases increases tumor cell apoptosis in HER2+ breast cancers
Source: Breast Cancer Res. 2015 Dec 4;17:148. doi: 10.1186/s13058-015-0656-2 (PMC4670529; doi:10.1186/s13058-015-0656-2)
Supplement: Additional file 1: Figure S1. — EZN4150 reduces expression of p110α without effecting p110β. (PDF 851 kb) [file 13058_2015_656_MOESM1_ESM.pdf]

SUPPLEMENTARY FIGURES

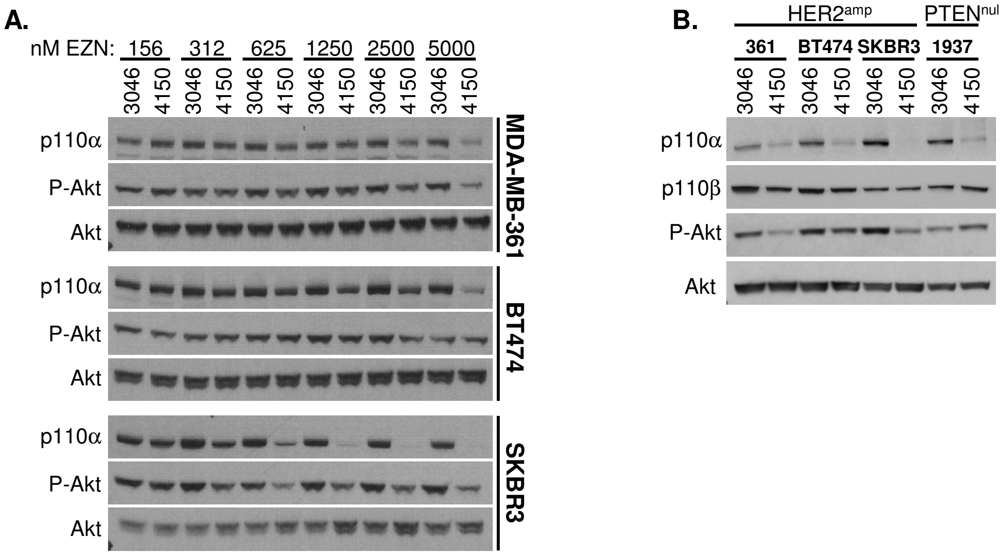

Supplementary Figure 1: EZN4150 reduces expression of p110α without effecting p110β: Cells were treated with EZN3046 (scrambled control LNA-ASO) or EZN4150 (p110α targeted LNA-ASO) for 10 days, with fresh media and LNA-ASOs replenished every three days. **(A)** Cells treated with the indicated concentration of compounds were lysed and the lysates were analyzed by immunoblot with the indicated antibodies. **(B)** MDA-MB-361 (361), BT474, SKRB3 or HCC1937 (1937) cells treated with the 5 μM EZN compounds were lysed and the lysates analyzed by immunoblot.
