# Supplementary material for: Dual inhibition of Type I and Type III PI3 kinases increases tumor cell apoptosis in HER2+ breast cancers
Source: Breast Cancer Res. 2015 Dec 4;17:148. doi: 10.1186/s13058-015-0656-2 (PMC4670529; doi:10.1186/s13058-015-0656-2)
Supplement: Additional file 2: Figure S2. — EZN4150 and p110α siRNA target sequences within the PIK3CA gene. (PDF 851 kb) [file 13058_2015_656_MOESM2_ESM.pdf]

```
EZN4150 (LNA antisense):           AGCCATTCATTCCACC
EZN4150 sense seqeuence target:    GGTGGAATGAATGGCT
PIK3CA siRNA #38 target sequence:  CTGAGTAACAGACTAGCTA
PIK3CA siRNA #39 target sequence:  CTTATTGAGGTGGTGCGAA
```

Human *PIK3CA* (NCBI Reference Sequence: NM_006218.2):

```
ATGCCTCCACGACCATCATCAGGTGAACTGTGGGGCATCCACTTGATGCCCCCAAGAATCCTAGTAGAATGTTTACTACCAAATGGAATGATAGTGAC
TTTAGAATGCCTCCGTGAGGCTACATTAATAACCATAAAGCATGAACTATTTAAAGAAGCAAGAAAATACCCCCTCCATCAACTTCTTCAAGATGAAT
CTTCTTACATTTTCGTAAGTGTTACTCAAGAAGCAGAAAGGGAAGAATTTTTTGATGAAACAAGACGACTTTGTGACCTTCGGCTTTTTCAACCCTTT
TTAAAAGTAATTGAACCAGTAGGCAACCGTGAAGAAAAGATCCTCAATCGAGAAATTGGTTTTGCTATCGGCATGCCAGTGTGTGAATTTGATATGGT
TAAAGATCCAGAAGTACAGGACTTCCGAAGAAATATTCTGAACGTTTGTAAAGAAGCTGTGGATCTTAGGGACCTCAATTCACCTCATAGTAGAGCAA
TGTATGTCTATCCTCCAAATGTAGAATCTTCACCAGAATTGCCAAAGCACATATATAATAAATTAGATAAAGGGCAAATAATAGTGGTGATCTGGGTA
ATAGTTTCTCCAAATAATGACAAGCAGAAGTATACTCTGAAAATCAACCATGACTGTGTACCAGAACAAGTAATTGCTGAAGCAATCAGGAAAAAAAC
TCGAAGTATGTTGCTATCCTCTGAACAACTAAAACTCTGTGTTTTAGAATATCAGGGCAAGTATATTTTAAAAGTGTGTGGATGTGATGAATACTTCC
TAGAAAAATATCCTCTGAGTCAGTATAAGTATATAAGAAGCTGTATAATGCTTGGGAGGATGCCCAATTTGATGTTGATGGCTAAAGAAAGCCTTTAT
TCTCAACTGCCAATGGACTGTTTTACAATGCCATCTTATTCCAGACGCATTTCCACAGCTACACCATATATGAATGGAGAAACATCTACAAAATCCCT
TTGGGTTATAAATAGTGCACTCAGAATAAAAATTCTTTGTGCAACCTACGTGAATGTAAATATTCGAGACATTGATAAGATCTATGTTCGAACAGGTA
TCTACCATGGAGGAGAACCCTTATGTGACAATGTGAACACTCAAAGAGTACCTTGTTCCAATCCCAGGTGGAATGAATGGCTGAATTATGATATATAC
ATTCCTGATCTTCCTCGTGCTGCTCGACTTTGCCTTTCCATTTGCTCTGTTAAAGGCCGAAAGGGTGCTAAAGAGGAACACTGTCCATTGGCATGGGG
AAATATAAACTTGTTTGATTACACAGACACTCTAGTATCTGGAAAAATGGCTTTGAATCTTTGGCCAGTACCTCATGGATTAGAAGATTTGCTGAACC
CTATTGGTGTTACTGGATCAAATCCAAATAAAGAAACTCCATGCTTAGAGTTGGAGTTTGACTGGTTCAGCAGTGTGGTAAAGTTCCCAGATATGTCA
GTGATTGAAGAGCATGCCAATTGGTCTGTATCCCGAGAAGCAGGATTTAGCTATTCCCACGCAGGACTGAGTAACAGACTAGCTAGAGACAATGAATT
AAGGGAAAATGACAAAGAACAGCTCAAAGCAATTTCTACACGAGATCCTCTCTCTGAAATCACTGAGCAGGAGAAAGATTTTCTATGGAGTCACAGAC
ACTATTGTGTAACTATCCCCGAAATTCTACCCAAATTGCTTCTGTCTGTTAAATGGAATTCTAGAGATGAAGTAGCCCAGATGTATTGCTTGGTAAAA
GATTGGCCTCCAATCAAACCTGAACAGGCTATGGAACTTCTGGACTGTAATTACCCAGATCCTATGGTTCGAGGTTTTGCTGTTCGGTGCTTGGAAAA
ATATTTAACAGATGACAAACTTTCTCAGTATTTAATTCAGCTAGTACAGGTCCTAAAATATGAACAATATTTGGATAACTTGCTTGTGAGATTTTTAC
TGAAGAAAGCATTGACTAATCAAAGGATTGGGCACTTTTTCTTTTGGCATTTAAAATCTGAGATGCACAATAAAACAGTTAGCCAGAGGTTTGGCCTG
CTTTTGGAGTCCTATTGTCGTGCATGTGGGATGTATTTGAAGCACCTGAATAGGCAAGTCGAGGCAATGGAAAAGCTCATTAACTTAACTGACATTCT
CAAACAGGAGAAGAAGGATGAAACACAAAAGGTACAGATGAAGTTTTTAGTTGAGCAAATGAGGCGACCAGATTTCATGGATGCTCTACAGGGCTTTC
TGTCTCCTCTAAACCCTGCTCATCAACTAGGAAACCTCAGGCTTGAAGAGTGTCGAATTATGTCCTCTGCAAAAAGGCCACTGTGGTTGAATTGGGAG
AACCCAGACATCATGTCAGAGTTACTGTTTCAGAACAATGAGATCATCTTTAAAAATGGGGATGATTTACGGCAAGATATGCTAACACTTCAAATTAT
TCGTATTATGGAAAATATCTGGCAAAATCAAGGTCTTGATCTTCGAATGTTACCTTATGGTTGTCTGTCAATCGGTGACTGTGTGGGACTTATTGAGG
TGGTGCGAAATTCTCACACTATTATGCAAATTCAGTGCAAAGGCGGCTTGAAAGGTGCACTGCAGTTCAACAGCCACACACTACATCAGTGGCTCAAA
GACAAGAACAAAGGAGAAATATATGATGCAGCCATTGACCTGTTTACACGTTCATGTGCTGGATACTGTGTAGCTACCTTCATTTTGGGAATTGGAGA
TCGTCACAATAGTAACATCATGGTGAAAGACGATGGACAACTGTTTCATATAGATTTTGGACACTTTTTGGATCACAAGAAGAAAAAATTTGGTTATA
AACGAGAACGTGTGCCATTTGTTTTGACACAGGATTTCTTAATAGTGATTAGTAAAGGAGCCCAAGAATGCACAAAGACAAGAGAATTTGAGAGGTTT
CAGGAGATGTGTTACAAGGCTTATCTAGCTATTCGACAGCATGCCAATCTCTTCATAAATCTTTTCTCAATGATGCTTGGCTCTGGAATGCCAGAACT
ACAATCTTTTGATGACATTGCATACATTCGAAAGACCCTAGCCTTAGATAAAACTGAGCAAGAGGCTTTGGAGTATTTCATGAAACAAATGAATGATG
CACATCATGGTGGCTGGACAACAAAAATGGATTGGATCTTCCACACAATTAAACAGCATGCATTGAACTGA
```

Supplementary Figure 2: EZN4150 and p110α siRNA target sequences within the *PIK3CA* gene: The human *PIK3CA* coding sequence is presented. The nucleotide sequences targeted by EZN4150, si*PIK3CA* #38 and si*PIK3CA* #39 are highlighted.
