# Supplementary material for: Dual inhibition of Type I and Type III PI3 kinases increases tumor cell apoptosis in HER2+ breast cancers
Source: Breast Cancer Res. 2015 Dec 4;17:148. doi: 10.1186/s13058-015-0656-2 (PMC4670529; doi:10.1186/s13058-015-0656-2)
Supplement: Additional file 3: Figure S3. — Combined inhibition of human epidermal growth factor receptor-2 (HER2), p110α and Vps34 induces cell death. (PDF 851 kb) [file 13058_2015_656_MOESM3_ESM.pdf]

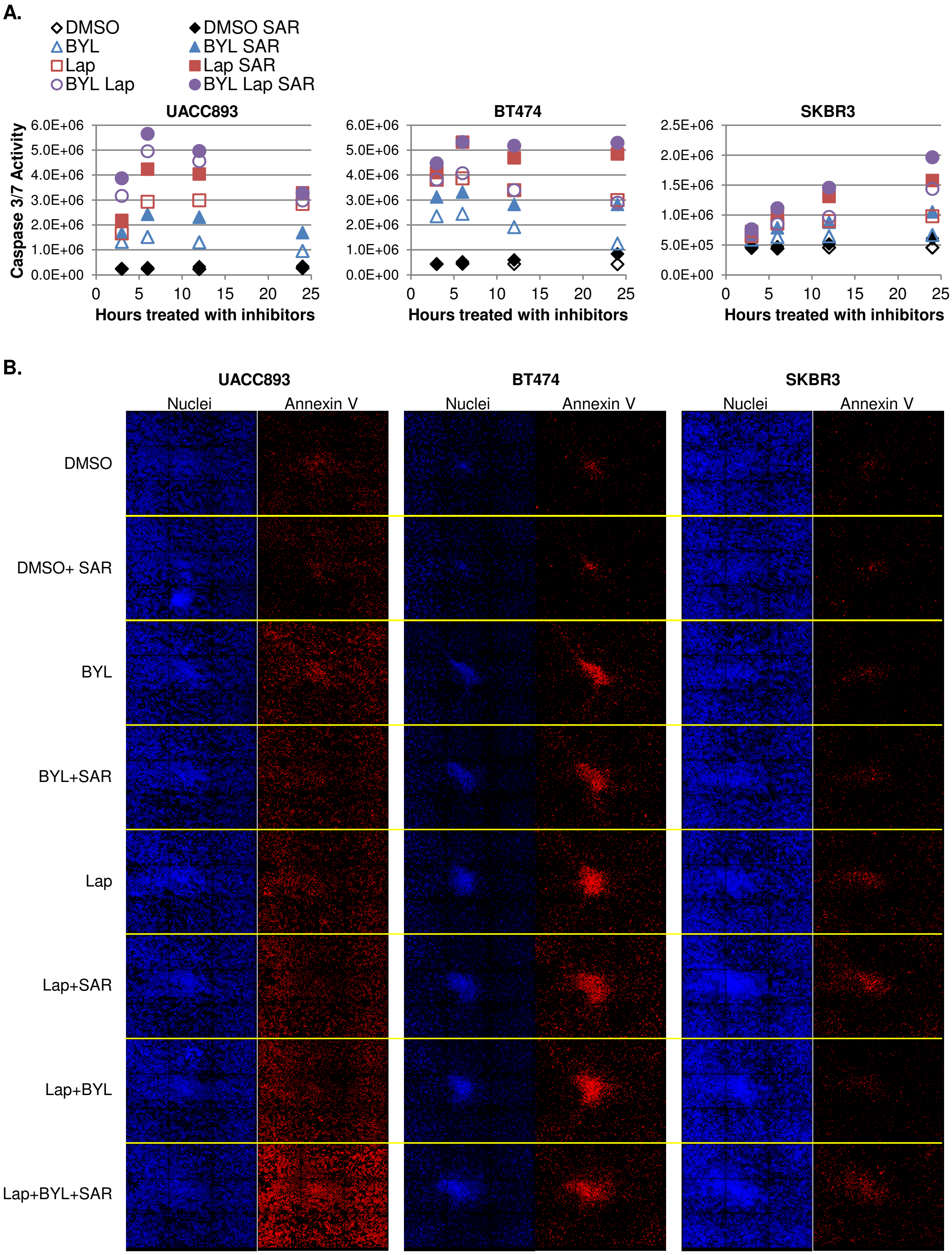

Supplementary Figure 3: Combined inhibition of HER2, p110α and Vps34 induces cell death: (A) UACC893, BT474 or SKBR3 cells were treated 3, 6, 12 and 24 h with 1 µM SAR405, 0.5 µM Lapatinib and/or 0.5 µM BYL719 before caspase 3/7 activity was determined. **(B)** UACC893 and BT474 were treated 6 h and SKBR3 were treated 24 h before being stained with Annexin V (red) with a nuclear counterstain (blue). 9 images were captured per well of quadruplicate wells. All 9 images of a representative well are presented. In both experiments, SAR405 was applied 30 min prior to the application of BYL719, lapatinib or DMSO control.
